# Actual Weight, Perceived Weight and Desired Weight of Romanian School Children by Parents and Children

**DOI:** 10.3390/medicina57040333

**Published:** 2021-04-01

**Authors:** Lucia Maria Lotrean, Ioana Popa, Mira Florea, Cecilia Lazea, Ana Maria Alexandra Stanescu, Codruta Lencu

**Affiliations:** 1 Department of Community Medicine, Iuliu Hatieganu University of Medicine and Pharmacy, 400012 Cluj-Napoca, Romania; llotrean@umfcluj.ro; 2 Dietician, Iuliu Hatieganu University of Medicine and Pharmacy, 400012 Cluj-Napoca, Romania; ioanapopand@yahoo.com; 3 Family Medicine Department, Iuliu Hatieganu University of Medicine and Pharmacy, 400012 Cluj-Napoca, Romania; 4 Department Pediatrics I, Emergency Pediatric Hospital, Iuliu Hatieganu University of Medicine and Pharmacy, 400012 Cluj-Napoca, Romania; cicilazearo@yahoo.com; 5 Departament of Family Medicine, Carol Davila University of Medicine and Pharmacy, 020021 Bucharest, Romania; alexandrazotta@yahoo.com; 6 Department of Endocrinology, Iuliu Hatieganu University of Medicine and Pharmacy, 400012 Cluj-Napoca, Romania; codruta_lmc@yahoo.com

**Keywords:** body weight, misperception, children, parents, weight management

## Abstract

*Background and Objectives:* The perception of the body weight by children and parents influences the consequent actions undertaken for children’s body weight management. This study investigated the correspondence between objective evaluations of Romanian school children (actual weight) and perceptions about weight (perceived weight), preoccupation with body weight management (desired weight) and parents’ perceptions on children’s weight. *Materials and Methods:* A cross sectional study was performed among 344 children aged 11 to 14 and 147 parents from Cluj-Napoca, Romania. We made anthropometric measurements of children, and short questionnaires were completed by the children and the parents. *Results:* The results show that 3.8% of children were underweight, 68.3% had a normal weight and 27.9% were overweight. Of this sample, 61.5% of underweight children, 20% of normal weight children and 43.7% of overweight children had misperceptions about their weight. The percentage of parents who did not estimate their children’s weight correctly was 50%, 11.9% and 41.5%, respectively, for each of the three weight groups. The results of the logistic regression analyses showed that several factors were associated with the misclassification of their own body weight by the children, such as body mass index, gender, weight management practices, misclassification by the parents as well as parent–child discussions on these issues. *Conclusions:* Education for both Romanian parents and children is needed with regard to correctly identifying and managing children’ body weight.

## 1. Introduction

Children’s body weight has important short and long-term consequences on their health, social and psychological development [1,2]. Underweight is mainly a consequence of inadequate diet and frequent infections, leading to deficiencies in calories, proteins, vitamins and minerals [3]. On the other hand, childhood overweight and obesity can be linked to several medical conditions such as metabolic, cardiovascular, orthopedic, neurological, hepatic, pulmonary, and renal disorders. Moreover, it can affect social and emotional health, as well as academic performance [1,2] especially since the overweight child is likely to continue to be overweight at adulthood [3].

The perception of the body weight by children and parents influences the consequent actions undertaken for the children’s body weight management [4,5,6,7,8,9,10]. The children’s perception of body weight determines the quantity and quality of food intake, body weight management practices and communication with parents, friends and health professionals on this issue [5,6,7]. The parents’ perception of the children’s nutritional status contributes to the pattern of care that includes in-house feeding practices, shaping daily eating habits, and seeking professional help when needed [8,9,10].

Misperceptions of the children’s body weight by both the children and the parents might lead to unhealthy eating habits and inappropriate actions for combating overweight or underweight, while there is also concern that focusing excessively on a child’s weight could foster the development of body image problems, mental health issues and disordered eating [6,7,8,9,10]. The challenge is to assess the children’s and parents’ perceptions of the real weight of the children and to correct misperceptions, applying educational interventions for appropriate children’s body weight management.

Studies performed in several countries that proved that misperceptions regarding body weight as well as body dissatisfaction among children is influenced by several individual, familial and social factors, such as residence, age, gender, socio-economic factors, lifestyle-related behaviors, mental wellbeing, familial opinions and practices related to eating habits and weight management, communication with peers and family, media messages, and health promotion actions, and might suffer differences between countries, making it important to perform studies which identify these issues in different population groups from different countries [9,10,11,12,13,14,15,16,17,18,19].

In Romania, several studies have focused on different groups of children’s and adolescents’ body weight measurement [18,19,20,21]. In 2017/18, study about health behavior in school aged children investigated the prevalence of overweight and obesity, as well as underweight and body image among school children aged 11, 13 and 15 from Romania, but no objective measurements were made, with the data relying on the children’s declarations regarding their own height and weight. The results showed that among girls, overweight (including obesity) was 22% (for 11-year-olds), 15% (both among 13- and 15-year-olds), while among boys the results were 32%, 30% and 27%, respectively, for the three age groups. Underweight varied between 4–6%, both among girls and boys of different ages. The percentage of girls who thought they were too fat varied between 24 % among 11-year-olds to 34% among 15-year-olds, while among boys the percentage was 25% among 11-year-olds, 24% among 13-year-olds and 21% among 15-year-olds [18].

To the best of our knowledge, no study has investigated yet the correspondence between the objective evaluation of the Romanian children’s weight and its perception by children and parents.

This study had three objectives. First, it aimed to determine the correspondence between anthropometric evaluations of Romanian children (actual weight) and their perceptions on weight (perceived weight) as well as their preoccupation with body weight management (desired weight). Second, it investigated the correspondence between the actual weight of Romanian children and the parents’ perception of children’s weight. Third, it assessed the differences between children who estimated their weight correctly and those who estimated it inappropriately.

## 2. Materials and Methods

### 2.1. Study Design and Sample

We performed a cross sectional study in 2 schools from Cluj-Napoca, a town with more than 300,000 inhabitants from north-west Romania in the period March-May 2013. Approval for the study was obtained in December 2012 from the review committees formed by school directors and teachers of the two schools, according to the standard procedure in Romania at that time. A member of the research team had meetings with the representatives of the schools, presented the objectives, characteristics and tools of the project, which entailed assessing children’s physical development through anthropometric measurements, as well as the evaluation through questionnaires of opinions and practices with regard to their body weight among school children and opinions regarding the body weight of their children among the children’s parents. The representatives of the schools gave permission and made the logistical arrangements for the implementation of the project in schools.

The sample included 344 school children (169 girls and 175 boys) aged 11–14 years from all classes from grades V to VII from two schools. All students present in the class during the days when the study was performed were included in study.

Children were informed that the study involved anthropometric measurements (weight and height) and filling in by them of short questionnaires. At the same time, it was clearly specified orally as well as in written at the beginning of the questionnaire that their participation was voluntary, while all the data would be confidential, with only the research team having access to the collected information. School children could refuse to participate by not filling in the questionnaire, while filling in the questionnaire was an informed consent for their participation. No refusal was recorded. All students filled in the questionnaires and after that the anthropometric measurements were performed.

The parents of the participating students were also invited to participate by filling in a questionnaire sent through letters. Similarly to the questionnaire for the children, it clearly specified the voluntary and confidential nature of data collection.

### 2.2. Instruments for Data Collection

#### Anthropometric Measurements

Children were evaluated through anthropometric measurements Height and weight were measured in the class by student dieticians. Weight was measured in minimal clothing, without shoes, to the nearest 0.1 kg, with an electronical step scale; height was measured to the nearest 0.5 cm in the same conditions using a stadiometer.

#### Questionnaires

Children were asked to fill in a short questionnaire assessing their demographics characteristics (age, gender, school) as well as opinions and practices with regard to their body weight:

- Perceived weight—children were asked how they considered their weight, the possible answers being normal, too low, too big. A similar approach was used by other studies, too [10,11,12,13,15,18,19].
- Weight management practices in the last year (attempts to lose weight, nothing or attempts to gain weight in the last year);
- Types of methods used in the last year for losing weight if they had tried to do this (children could choose from sport, dieting, consumption of special tea, use of medicine, vomiting, and massage);
- If they discussed about their weight with their parents in the last year (yes/no)
- If they discussed issues focused on their weight with health professionals in the last year (yes/no)

Short questionnaires for parents were sent and returned through letters delivered through the children. The questionnaires assessed the opinions of parents regarding the weight of their children—perceived weight (normal, too low, too big)—and if they discussed issues related to their children’s weight with health professionals in the last year.

### 2.3. Data Analyses

Body mass index (BMI) was calculated from weight and height measurements, based on the following formula: BMI = weight (kg)/height (m)^2^. Normal nutritional status, underweight, overweight, obesity were established based on the World Health Organization (WHO) recommendations regarding BMI for age and sex (z-scores). Overweight: > +1SD (equivalent to BMI 25 kg/m^2^ at 19 years); obesity: > +2SD (equivalent to BMI 30 kg/m^2^ at 19 years); thinness: < −2SD [22].

The prevalence of the investigated issues was calculated. Correct/incorrect estimation of actual weight was estimated as follows:

- If children with normal BMI declared that they considered their weight normal they had correct estimation; if they said it was too big or too little they had incorrect estimation (overestimation, underestimation, respectively).
- If children with overweight/obesity said that their weight was too high they had correct estimation, if they considered it normal they had incorrect estimation (underestimation).
- If underweight children said their weight was too little they had correct estimation, if they said it is normal they had incorrect estimation (overestimation).

Logistic regression analysis was performed in order to assess the factors associated with higher BMI (coded as 0—normal weight, 1—overweight, including obesity), with the independent variables being age, gender, estimation of children’s weight by children and parents, respectively (both variables were coded as 0—incorrect estimation, 1—correct estimation), the presence of attempts to decrease/increase their weight in the last year (0—no, 1—yes), the presence of discussions between children and parents, respectively, and health professionals with regard to their weight, as well as between parents and health care professionals (0—absent, 1—present).

On the other hand, logistical regression analyses were also used in order to assess differences between children with normal weight who estimated their weight correctly and those who underestimated or overestimated, it as well as between children with overweight who correctly estimated and those who underestimated their weight. Dependent variables were coded as 0—incorrect estimation (overestimation or underestimation), 1—correct estimation. The independent variables for all these logistic regression analyses were age, gender, the presence of attempts to decrease/increase their weight in the last year (0—no, 1—yes), the presence of discussions between children and parents, respectively, with health professionals with regard to their weight, as well as between parents and health care professionals (0—absent, 1—present), estimation of children weight by parents (coded as 0—incorrect estimation, 1—correct estimation). Due to the small sample, a similar analysis was not possible among children with underweight.

Statistical analyses were performed using IBM SPSS Statistics for Windows Version 20 program. Statistically significant results are reported at *p* < 0.05.

## 3. Results

### 3.1. Actual Weight, Perceived Weight and Desired Weight among School Children

The objective measurements of body weight showed that out of the school children, 3.8% were underweight, 68.3% had a normal weight and 27.9% were overweight (including obesity) (see Table 1).

Regarding perceived weight, only 38.5% of the underweight children recognized this, while the others considered they had a normal weight. Among normal weight children, the majority correctly estimated their weight, 11.1% considered they were underweight and 8.9% believed there are overweight. The percentage of overweight children who correctly estimated their weight was 56.2%, while the others thought their weight was normal.

The desired weight results showed that 61.5% of the underweight and 17.9% of the normal weight children tried to gain weight in the last year, while 82.7% of the overweight children and 31.9% of the normal weight children tried to lose weight in the last year. The respective methods used for losing weight among overweight children and children who had normal weight and declared attempts to lose weight in the last year were as follows: sport (80.1% and 84%), dieting (52.6% and 42.7%), consumption of special tea (3.2% and 0%), use of medicine (1.9% and 1.3%), vomiting (0.6% and 0%), and massage (1.3% and 0%).

The percentage of children who declared talking with their parents in the last year about their weight increased from 58.3% among underweight children to 73.2% among normal weight children and 84.7% among overweight children. The discussion with a health care professional in the last year concerning their weight was reported by 38.5% of the underweight children, 32.3% of the normal-weight children and 43.9% of the overweight children.

A total of 147 parents of the investigated children (85% women and 15% men) filled in the questionnaire. The parents correctly appreciated the weight of their children in half of the cases of underweight children, 88.1% of normal weight children and 59.5% of overweight children (see Table 1). Half of the parents of underweight children and around 40% of the overweight children misperceived their child’s weight as normal. Among normal weight children’s parents, 9.9% misperceived their child’s weight as too low and 2% appreciated it as too high.

Around half of the parents of children with underweight or normal weight and two thirds of parents having overweight children reported discussions with a physician in the last year focused on the child’s weight.

### 3.2. Factors Associated with Overweight

Table 2 shows that in comparison with children with a normal BMI, those with a higher BMI were more frequently boys and had a tendency to underestimate their weight, while their parents also did this. At the same time, they declared more frequent attempts to lose weight in the last year, and had more frequently discussed about their weight with their parents and health professionals in the last year, while their parents were more eager to bring up this issue during discussions with health professionals.

### 3.3. Factors Associated with Correct, Underestimation and Overestimation of Their Own Weight among Romanian Children

A percentage of 28.2% of the children had an incorrect estimation of their own weight, while 21.1% of the parents had misperceptions regarding their children’s weight.

As Table 2 shows, in comparison with normal weight children who misperceived their weight as low, normal weight children who correctly estimated their weight were more frequently girls and were less likely to declare attempts to gain weight as well as less likely to report that they talked with their parents about their weight; correct estimation of their own weight by the children was associated with higher correct estimation of their children’s weight by parents.

On the other hand, in comparison with normal weight children who misperceived themselves as having a higher weight than their actual one, normal weight children who correctly estimated their weight were more frequently boys and were less likely to attempt weight loss, but no difference was found between the two groups with regard to the perceptions of their parents (see Table 2).

Table 2 presents that, among overweight children, in comparison with those who incorrectly considered their weight as normal, the children who correctly estimated their weight were more likely to try to lose weight and to talk with their parents about this issue. At the same time, the correct estimation of their own weight by overweight children was associated with higher correct estimation of the children’s weight by their parents.

## 4. Discussion

This study focuses on assessing the actual weight, as well as perceived weight and desired weight of Romanian school children. The results show that more than one quarter of the children are overweight, while only 4% are underweight, with boys having a higher risk for being overweight. The results are similar to those from other Romanian studies which used anthropometric measurements [20,21], and one has evidenced the increasing prevalence trend of overweight children in a pooled analysis of cross-sectional studies between 2006 and 2015 [21]. The prevalence of underweight children was similar to that in our study (4%), while that of overweight (including obese) children was 28.3%. Male gender compared to female, prepubertal age compared to post pubertal age and urban environment had higher risk for overweight [21]. A survey performed in Bucharest, the capital of Romania, among school children aged 6–18 showed a prevalence of 31.6% overweight (including obese) based on WHO standards [20].

Research in the field of the lifestyles of children is time and energy consuming process, but definitely also a rewarding one, since the results are important pillars for designing appropriate health promotion interventions [23]. In Romania, no study has yet investigated the correspondence between the objective evaluation of secondary school children’s body weight and its perception by children and parents. The results of our study showed that 28.2% of the children had an incorrect estimation of their own weight, with overweight children being more prone to have incorrect estimation of their own weight in comparison with the children with normal weight. Studies performed in United States of America, Australia and other European countries showed a prevalence of incorrect estimation of their own weight by children and adolescents varying between 29% and more than 40%, depending on the country, age and BMI [11,12,13,14,15]. Several studies also showed a higher tendency of overweight children than normal weight children to misclassify their own weight [11,12,13].

In our study, we identified several misperceptions regarding their weight among all three BMI groups. The percentage of underweight children was low, but two thirds of them considered their weight as normal. Nevertheless, two thirds of the underweight children have tried to gain weight in the last year, showing a preoccupation with having a higher desired weight. It might be possible that these attempts led to a certain increase in weight and, because of missing appropriate knowledge and skills to correctly appreciate the weight, this made children think that their weight had reached normal levels.

Among normal weight children, the majority recognize their real weight, but one out ten children misperceived it as overweight and one third reported that they had tried to lose weight in the last years. This misperception was more frequent among girls and was associated with a greater tendency to try to lose weight. These data show the tendency of some children to have unrealistic expectations regarding the need to lose weight, either because they misjudge their weight or because they want to lose weight, despite the fact that they see their weight as normal. It is probably a consequence of several media and peer messages portraying beauty through images of slim persons and encouraging the idea of being slim as a socially desired norm [24,25,26,27].

On the other side, one in ten normal weight children think their weight is too low, while 18% of the children have tried to gain weight in the last year. Children with this misperception were more likely to try to gain weight. This situation could be the consequence of the fact that children do not have the ability to appreciate the optimal weight and could do so by visual comparisons with other children, while in several Romanian families, parents have constant concerns about feeding their children enough food, without sufficient knowledge of what the proper nutrition of their children means in terms of food quantity and quality [19].

Almost half (43.8%) of the overweight children did not recognize their abnormal nutritional status, thinking that their weight was normal. No age or gender differences were found with regard to correct estimation of their own weight among overweight children. Nevertheless, 82% of the overweight children in our study have tried to lose weight, mainly by sport and dieting, with overweight children who correctly estimated their weight being more likely to do this. The study did not investigate in detail the change in the body weight after these attempts to lose weight, or if the attempts were correct, serious and with results, but the data underlines that these students need to have a structured health promotion program which educates them on correct nutrition and exercise habits, supports them to set and work towards realistic goals with regard to losing weight, and teaches them how to maintain their real progress.

At the same time, our study showed that 21.1% of the parents did not correctly estimate the weight of their children; again, this situation was more frequent among the parents of overweight children in comparison with those with normal weight. Studies from other studies highlighted that parents’ misperceptions regarding the weight of their children vary depending on country/region, their children’s age group, the actual weight of children and the time when the study was performed, with several studies showing that these misperceptions are more likely among the parents of overweight children [28,29,30,31,32,33,34]. For instance, a large study from Australia showed that one quarter of mothers misclassified the weight of their 14 years old children, while a—country European study showed that 27.6% of parents of overweight/obese children underestimated their children’s weight status [5,7].

Parents’ underestimation of their child’s weight is due to the fact that some of them do not have a clear definition of what overweight means—they might have limited information from health professionals or even tend not to believe in physician/clinical charting of weight and BMI percentiles and estimate the weight through visual comparison with other children Another reason for weight status misclassification could be that the parents are aware of their child’s weight, but due to social undesirability, they do not wish to label their children as overweight [5,34]. There are some parents who recognize their child’s weight, but others may feel embarrassed to discuss their child’s overweight or obesity and may feel reluctant in seeking the advice of a health care professional [5,34]. In our study, both among normal weight and overweight children, those who correctly estimated their weight also more frequently had parents who did so in comparison with those who underestimated their weight.

Communications between children and parents, as well as between children and parents and health professionals with regard to children’s weight is an important step in order to identify and correct misperceptions and adopt healthy lifestyle behaviors which contribute to the prevention and correction of underweight and overweight [32,35]. In our study all these forms of communication were more frequent among overweight children in comparison with normal weight children. Moreover, among normal weight children the discussions with parents were reported more frequently by children with incorrect underestimation of their weight, while among overweight children the discussion with parents on this issue were more frequent among those who correctly estimated that their weight was too high. Similarly to studies from other countries, these data suggest that if parents fail to correctly estimate the weight of their children, it is unlikely they will recognize the importance of interventions targeting the promotion of healthy nutrition and body weight management [5,32,33,34,35].

The current epidemiological context with online schooling, inadequate physical activity and sedentary behavior in response to COVID-19 pandemic, may lead to an increased risk of childhood obesity [36]. Although data collection from our study predates the COVID-19 pandemic, these results and subsequent parent and child-based educational interventions, reset in this long-term epidemiological context, could positively influence children’s COVID-related weight gain due to the switch to online learning and improve their nutritional status recognition. Healthcare professionals should share their recommendations with both parents and children for weight gain control, as well as interventions focused on increasing the accuracy of normal weight perception, helping to promote healthy weight loss and maintaining optimal weight [35,36].

This study is subject to several limitations. First, it included a sample of children aged 12–14 from one big city of Romania, but because of logistical and funding constraints it did not include a nationally representative sample, so the generalization of these results beyond this sample is limited Second, similarly to other studies, this is an exploratory study assessing the perceived weight of children based on a question which investigated if children believed their weight was normal, too low or too high; future studies might use more complex scales for assessing this issue. Third, the participation rate of the parents was modest, while the results are based only on the information declared by both parents and children through means of short questionnaires, since the study is an exploratory one. Future studies should try to motivate the parents to participate more and should focus more on details regarding the knowledge, attitudes and practices regarding body weight management both among children and parents. Last, but not least, the study was performed in 2013 and in the last few years, especially during the COVID-19 pandemic, several changes might have happened with regard to opinions and practices regarding weight and weight management both among children and parents in Romania. Nevertheless, this study investigated for the first time in Romania the correspondence between actual weight, perceived weight and desired weight among children and their parents, offering information needed for the activities of health promotion in this field. Moreover, the data made available by this study could be useful for the better assessment and understanding of the changes with regard to this subject over a longer period of time, if future studies should happen to investigate these issues.

## 5. Conclusions

Our study identified children’s and parents’ misperceptions about children’s weight, having as consequences a lack of parent–child communication about health issues, unhealthy practices for the self-management of weight, delayed addressing of weight-related issues to healthcare professionals and late interventions.

Efforts should focus on developing school based programs which assess children’s weight and communicate correctly to both children and parents their nutritional status, as well as actions which should be taken for the appropriate management of children’s weight and childhood health promotion. Further studies are needed to explore educational strategies dedicated to both parents and children, focused on optimal recognition and proper management of children’s body weight.

## Figures and Tables

**Table 1:** Actual weight, perceived weight and desired weight among Romanian school children.

	Actual Weight		
	Underweight	Normal Weight	Overweight
	*n* = 13%	*n* = 235%	*n* = 96%
**Perceived weight by children**			
Underweight	38.5	11.1	0.0
Normal weight	61.5	80.0	43.7
Overweight	0.0	8.9	56.3
**Desired weight by children**			
Attempts to lose weight	0.0	31.9	83.3
Attempts to gain weight	61.5	17.9	0.0
None of the above	38.5	50.2	16.7
**Discussion between children and health care professionals**	38.5	32.3	42.7
**Discussion between children and** **parents**	58.3	73.2	85.4
**Perceived weight by parents**			
Underweight	50	9.9	0.0
Normal weight	50	88.1	41.5
Over weight	0.0	2.0	58.5
**Discussions between parents and health care professionals**	50	48.5	68.3

**Table 2:** Factors associated with BMI, correct, underestimation and overestimation of their own weight among Romanian children ^a.^

	BMI	Normal Weight Children		Overweight Children
	Overweight vs. Normal Weight OR (95%CI) ^b^	Correct Estimation vs. Incorrect Underestimation OR (95%CI) ^c^	Correct Estimation vs. Incorrect Overestimation OR (95%CI) ^d^	Correct Estimation vs. Incorrect Underestimation OR (95%CI) ^c^
**Gender**				
Girls	0.59 (0.37–0.96) *	2.5 (1.03–6.03) *	0.11 (0.03–0.51) **	1.60 (0.69–3.69) •
Boys	1	1	1	1
**Age**	0.95 (0.71–1.27) •	0.75 (0.45–1.24) •	0.61 (0.34–1.08) •	0.91 (0.55–1.62) •
**Weight estimation by children**				
Correct	0.32 (0.19–0.53) ***	-	-	-
Incorrect	1			
**Attempts to gain weight in the last year ^e,f^**				
**Yes**	-	0.05(0.01–0.13) ***	-	-
**No**		1		
**Attempts to lose weight in the last year ^g^**				
**Yes**	10.16(5.63–18.34) ***	-	0.24(0.09–0.62) **	7.62(2.00–29.97) **
**No**	1		1	1
**Discussion between children and parents**				
Yes	2.02 (1.08–3.77) *	0.21 (0.05–0.95) *	1.96(0.78–4.92) •	3.90(1.12–13.51) *
No	1	1	1	1
**Discussion between children and medical professionals**				
Yes	1.63 (1.01–2.65) *	0.95 (0.40–2.25) •	2.14(0.69–6.63) •	1.40(0.61–3.18) •
No	1	1	1	1
**Weight estimation by parents**				
Correct	0.19 (0.08-0.46) ***	10.1 (2.34–40.79) **	2.58 (0.44–14.98) •	7.20 (1.78–29.01) **
Incorrect	1	1	1	1
**Discussion between parents and medical professionals**				
Yes	2.12 (1.01–4.49) *	0.45 (0.12–1.62) •	1.44 (0.43–4.80) •	2.88 (0.74–11.20) •
No	1	1	1	1

^a^ The table depicts the results of logistic regression analyses; ^b^ BMI coded as 0-normal, 1- overweight; ^c^ Coded as 0-incorect underestimation, 1- correct estimation; ^d^ Coded as 0-incorect overestimation, 1-correct estimation; ^e^ Overweight children did not declare attempts to gain weight in the last year; ^f^ Normal weight children who considered their weight to be high did not declare attempts to gain weight in the last year; ^g^ Normal weight children who considered their weight to be low did not declare attempts to lose weight in the last year; • *p* > 0.05; * *p* < 0.05; ** *p* < 0.01; *** *p* < 0.001.

## Data Availability

The data presented in this study are available on request from the first author.
